# Differentially Expressed Genes Associated with the Cabbage Yellow-Green-Leaf Mutant in the *ygl-1* Mapping Interval with Recombination Suppression

**DOI:** 10.3390/ijms19102936

**Published:** 2018-09-27

**Authors:** Xiaoping Liu, Hailong Yu, Fengqing Han, Zhiyuan Li, Zhiyuan Fang, Limei Yang, Mu Zhuang, Honghao Lv, Yumei Liu, Zhansheng Li, Xing Li, Yangyong Zhang

**Affiliations:** Institute of Vegetables and Flowers, Chinese Academy of Agricultural Sciences, Key Laboratory of Biology and Genetic Improvement of Horticultural Crops, Ministry of Agriculture, Beijing 100081, China; 82101181120@caas.cn (X.L.); yuhailong@caas.cn (H.Y.); feng857142@163.com (F.H.); 82101181071@caas.cn (Z.L.); fangzhiyuan@caas.cn (Z.F.); yanglimei@caas.cn (L.Y.); zhuangmu@caas.cn (M.Z.); lvhonghao@caas.cn (H.L.); liuyumei@caas.cn (Y.L.); lizhansheng@caas.cn (Z.L.); xzdlixing@126.com (X.L.)

**Keywords:** cabbage, yellow-green-leaf mutant, recombination-suppressed region, bulk segregant RNA-seq, differentially expressed genes

## Abstract

Although the genetics and preliminary mapping of the cabbage yellow-green-leaf mutant YL-1 has been extensively studied, transcriptome profiling associated with the yellow-green-leaf mutant of YL-1 has not been discovered. Positional mapping with two populations showed that the yellow-green-leaf gene *ygl-1* is located in a recombination-suppressed genomic region. Then, a bulk segregant RNA-seq (BSR) was applied to identify differentially expressed genes (DEGs) using an F_3_ population (YL-1 × 11-192) and a BC_2_ population (YL-1 × 01-20). Among the 37,286 unique genes, 5730 and 4118 DEGs were detected between the yellow-leaf and normal-leaf pools from the F_3_ and BC_2_ populations. BSR analysis with four pools greatly reduced the number of common DEGs from 4924 to 1112. In the *ygl-1* gene mapping region with suppressed recombination, 43 common DEGs were identified. Five of the DEGs were related to chloroplasts, including the down-regulated *Bo1g087310*, *Bo1g094360,* and *Bo1g098630* and the up-regulated *Bo1g059170* and *Bo1g098440*. The *Bo1g098440* and *Bo1g098630* genes were excluded by qRT-PCR. Hence, we inferred that these three DEGs (*Bo1g094360*, *Bo1g087310,* and *Bo1g059170*) in the mapping interval may be tightly associated with the development of the yellow-green-leaf mutant phenotype.

## 1. Introduction

Yellow-green-leaf mutants have been extensively studied in many species, including *Arabidopsis thaliana* [1], barley [2], *Brassica napus* [3], rice [4,5,6], cabbage [7], and muskmelon [8]. Leaf color mutants are an ideal model for studying mechanisms of photosynthesis and light morphogenesis, since yellow-green-leaf mutants are commonly related to chlorophyll synthesis or degradation [9,10].

Chlorophyll is the most important pigment related to photosynthesis. In *Arabidopsis*, 27 genes involved in 15 steps in the pathway from glutamyl-tRNA to chlorophylls a and b have been identified. Leaf color mutants commonly result from blocking a portion of the chlorophyll synthesis pathway, such as the synthesis of 5-aminolevulinic acid (ALA) [11]. Runge et al. [12] isolated and classified some chlorophyll-deficient xantha mutants of *Arabidopsis thaliana* and found that some of the mutants were blocked at various steps of the chlorophyll pathway between ALA and protochlorophyllide (Pchlide), and the latter did not accumulate in the dark.

Bulked segregant analysis (BSA) is a powerful strategy that is commonly used in gene mapping [13]. Futschik and Schlötterer showed that sequencing of pools of samples from individuals are often more effective for Single Nucleotide Polymorphisms (SNP) discovery and provide more accurate allele frequency estimates [14]. Typically, two populations are used for BSA: a backcross (BC) population [15,16] and an F_2_ population [17,18]. Mackay and Caligari [19] found that quantitative trait loci (QTLs) are more easily detected in BC populations than in F_2_ populations.

In recent years, transcriptome analysis based on deep RNA sequencing (RNA-seq) has been used for the estimation of genome-wide gene expression levels [20,21]. Transcriptome sequencing encompasses mRNA transcript expression analysis. Combined RNA-seq analysis can be used for purposes such as novel transcript prediction, gene structure refinement, alternative splicing analysis, and SNP/InDel analysis [22]. Bulk segregant RNA-seq (BSR) has been applied to identify differentially expressed genes (DEGs) and trait-associated SNPs [23,24]. 

A yellow-green-leaf mutant (YL-1) was discovered in cabbage [10], and measurements of photosynthetic pigment contents, chloroplast ultrastructure, and chlorophyll fluorescence parameters indicated that YL-1 was deficient in its total chlorophyll content [10]. In a previous study, we mapped *ygl-1*, which controls the yellow-green-leaf phenotype, to chromosome C01 [7]. The linkage distance of the mapping interval was only 0.75 cM, but the physical distance in the reference genome TO1000 was ~10 Mb, indicating that recombination suppression existed in this interval. In this study, the recombination-suppressed region was identified by gene mapping. Two runs of BSR were performed using BC and F_3_ populations, with the aim of obtaining DEGs associated with the yellow-green-leaf mutant. 

## 2. Materials and Methods

### 2.1. Plant Materials

Group I: The F_2_, BC_1_P_1_, and F_3_ populations were constructed using as parents the yellow-green-leaf cabbage mutant YL-1 (P_1_) and the normal green leaf cabbage inbred line 01-20 (P_2_). The F_2_, BC_1_P_1_ population was employed for *ygl-1* mapping.

Group II: The BC_1_P_1_ and BC_2_P_1_ populations were constructed using as parents the mutant YL-1 (P_1_) and the normal green leaf Chinese kale inbred line 11–192 (P_3_) (Supplementary Figure S1). The BC_2_P_1_ population was employed for *ygl-1* mapping.

The F_3_ population in group I and the BC_2_ population in group II were used for RNA-seq analysis. All plant materials came from the Cabbage and Broccoli Research Group, the Institute of Vegetables and Flowers (IVF), and the Chinese Academy of Agricultural Sciences (CAAS).

### 2.2. Identification of Recombination Suppression in the ygl-1 Gene-Mapping Interval

**Figure 1:**
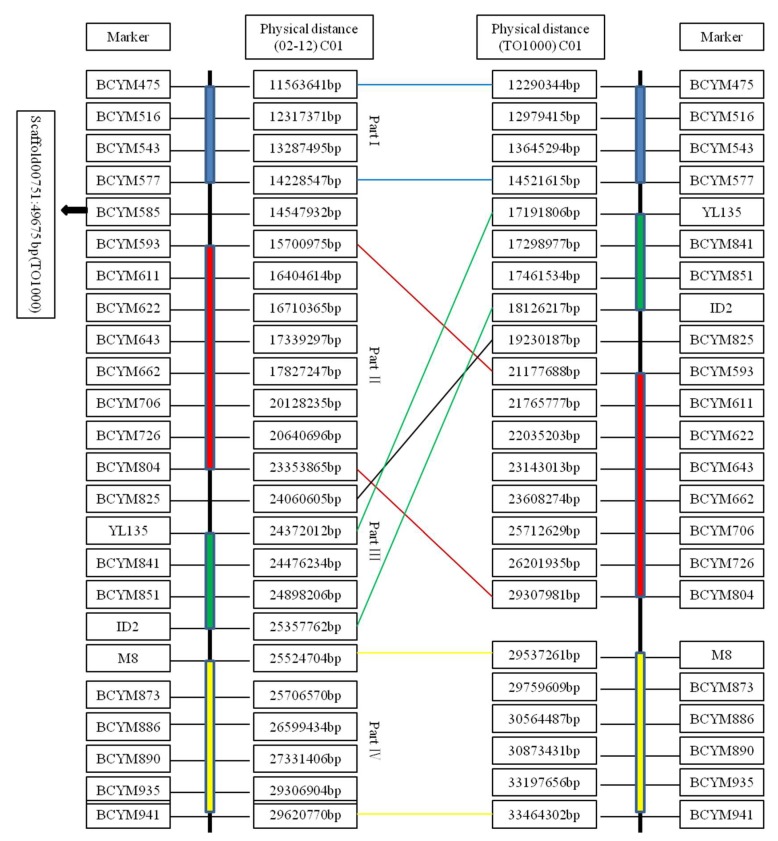
The physical distances of 24 InDel markers in the two reference genomes (02-12 and TO1000).

The sequences of 24 markers from the 02-12 reference genome (Supplementary Table S1) were aligned to chromosome C01 and the scaffold of the TO1000 reference genome [25] (Figure 1). Based on this alignment, we propose that possible assembly errors might exist in the 02-12 reference genome. Hence, InDel primers designed based on the TO1000 reference genome were applied for further mapping. The rates of recombination in the two populations were compared with the normal level in the cabbage genome (~600 kb/cM) to analyze the recombination-suppressed region.

### 2.3. BSA, RNA Isolation, and Library Construction

Before RNA isolation, leaf samples from the two populations (the F_3_ population in group I and the BC_2_ population in group II) were harvested to prepare four bulk groups: Bulk F_yellow (consisting of equal amounts of leaf tissues from 20 yellow-green-leaf F_3_ individuals), Bulk F_normal (20 normal-green-leaf F_3_ individuals), BC_yellow (20 yellow-green-leaf BC_2_ individuals), and BC_normal (20 normal-green-leaf BC_2_ individuals).

Total RNA extraction was performed according to instructions of the manufacturer of the TIANGEN kit employed for extraction (Invitrogen, Carlsbad, CA, USA). RNA purity was determined using a NanoDrop spectrophotometer (Thermo Fisher Scientific Inc., Wilmington, DE, USA), 1% formaldehyde gel electrophoresis, and a 2100 Bioanalyzer (Agilent Technologies, Santa Clara, CA, USA). 

A total amount of 1 μg of RNA per sample was employed for RNA sample preparation. Sequencing libraries were generated using the NEBNext^®^ Ultra^TM^ RNA Library Prep Kit for Illumina^®^ (Illumina, CA, USA) following the manufacturer’s recommendations. The cDNA library products were sequenced in a paired-end flow cell using an Illumina HiSeq^TM^ 2000 system.

## 3. Data Analysis

Reads containing adaptor sequences, low-quality reads (bases with more than 50% of quality scores ≤5), and unknown bases (>5% N bases) were removed from each dataset to obtain more reliable results, because such data negatively affect bioinformatics analyses. The sequencing reads were then aligned to the reference database for the *B. oleracea* genome (TO1000) (http://plants.ensembl.org/Brassica_oleracea/Info/Index) (accessed on 5 May 2017) [25] using HISAT [26]. Differential expression analysis to identify DEGs was performed using DESeq [27], with a threshold q value (or false discovery rate [FDR]) < 0.01 & |log_2_(fold change)| > 1 for significant differential expression. DEGs were displayed using Circos v0.66 [28]. GO (http://www.geneontology.org/) (accessed on 7 May 2017) [29] enrichment analysis of the DEGs was implemented using GOseq, in which gene length bias was corrected. GO functional analysis provides GO functional classifications and annotations for DEGs. Various genes usually cooperate with each other to exercise their biological functions. A pathway-related database was therefore obtained based on Kyoto Encyclopedia of Genes and Genome (KEGG) results (http://www.genome.jp/kegg/) (accessed on 11 May 2017) [30].

### Gene Expression Validation

DEGs associated with the yellow-green-leaf mutant were subjected to quantitative real-time RT-PCR (qRT-PCR) analysis. The primers designed according to the gene CDS sequences using DNAMAN are listed in Supplementary Table S6. Three technical replicates were performed for each gene. First-strand cDNA was synthesized using the PrimeScript^TM^ RT reagent Kit (TAKARA BIO, Inc., Shiga, Japan). qRT-PCR was performed with the SYBR Premix Ex Taq™ Kit (Takara, Dalian, China) with the following cycling parameters: 95 °C for five min, followed by 40 cycles of 95 °C for 10 s and 55 °C for 30 s, with a final cycle of 95 °C for 15 s, 55 °C for 60 s, and 95 °C for 15 s. Relative transcription levels were analyzed using the 2^−ΔΔ*C*t^ method [31]. qRT-PCR was performed in a BIO-RAD CFX96 system (Bio-Rad, Hercules, CA, USA), and the actin gene was employed as the internal control [32].

## 4. Results

### 4.1. Identification of the Recombination-Suppressed Region

In a previous study [7], we mapped *ygl-1,* which controls the yellow-green-leaf phenotype, to chromosome C01 using a population derived from YL-1 and 01-20. The *ygl-1* gene is flanked by the InDel markers ID2 and M8, and the interval between these two markers is 167 kb (C01: 25,357,762–25,524,704 bp) in the 02-12 reference genome. 

However, these two markers are anchored to the TO1000 reference genome, in which the interval between ID2 (C01: 18,126,217 bp) and M8 (C01: 29,537,261 bp) is 11.41 Mb, which is approximately 680 times greater than the distance (167 kb) in the 02-12 reference genome. Then, 24 markers from the 02-12 reference genome (Supplementary Table S1) were aligned to chromosome C01 and the scaffold of the TO1000 reference genome (Figure 1). In the 02-12 reference genome, the physical interval between BCYM475 (11,563,641 bp) and BCYM941 (29,620,770 bp) could be divided into four parts [Part I: BCYM475 (11,563,641 bp) to BCYM577 (14,228,547 bp); Part II: BCYM593 (15,700,975 bp) to BCYM804 (23,353,865 bp); Part III: YL135 (24,372,012 bp) to ID2 (25,357,762 bp); and Part IV: BCYM873 (25,706,570 bp) to BCYM941 (29,620,770 bp)]. The physical locations of Part I and Part IV in the two reference genomes were parallel. However, the physical locations of Part II and Part III were opposite. The makers’ order of linkage map was consistent with the physical map order of TO1000 reference genome but not 02-12 reference genome. Therefore, we proposed that an assembly error might exist in the 02-12 reference genome.

InDel primers designed based on the TO1000 reference genome were then applied for further mapping of the *ygl-1* gene. A total of 43 of the 62 pairs of InDel primers designed based on the TO1000 reference genome exhibited polymorphisms according to the F_3_ population. The genetic distances of the 16 InDel markers are shown in Table 1 (the sequences of these 16 markers are provided in Supplementary Table S2). The *ygl-1* gene was flanked by the InDel markers T1-36 (18,069,792 kb) and T1-58 (29,537,314 kb), with genetic distances of 0.42 cM and 0.42 cM, respectively. The interval distance between the two markers was 11.47 Mb based on the TO1000 reference genome. In the mapping region, spanning a physical distance of 11.47 Mb with a genetic difference of only 0.84 cM, the recombination rate was almost twenty times lower than the normal level for the cabbage genome (~600 kb/cM), suggesting that recombination suppression existed in this region. 

Another BC_2_P_1_ population, constructed with YL-1 and 11–192, was used to further identify recombination suppression. The *ygl-1* gene was flanked by InDel markers T1-34 (17,301,717 kb) and T1-58 (29,537,314 kb), with genetic distances of 0.3 cM and 0.7 cM, respectively. This result further demonstrated the existence of a recombination-suppressed region in the *ygl-1* mapping interval.

In a previous study [7], we showed that the region between markers the BCYM585 (14,547,932 bp) and BCYM825 (24,060,605 bp) exhibits recombination suppression. In Figure 1, the sequence of BCYM585 was aligned to an unanchored scaffold (Scaffold00751), and the sequence of BCYM825 was aligned to a physical distance of 19,230,187 bp based on the TO1000 reference genome. Part II was aligned between 21,177,688 bp and 29,307,981 bp based on the TO1000 reference genome. These results showed that the recombination-suppressed region observed between the markers T1-36 (18,069,792 kb) and T1-58 (29,537,314 kb) in this study was consistent with the recombination-suppressed region between the markers BCYM585 and BCYM825 identified in our previous study [7]. 

### 4.2. BSR Analysis, DEGs between the Yellow-Green-Leaf and Normal-Leaf Pools

BSR was applied to obtain DEGs using the F_3_ segregated population constructed with YL-1 and 01-20 and the BC_2_ population constructed with YL-1 and 11-192. A total of 339,481,468 reads were generated from the four cDNA libraries. Among these reads, 82,143,852 were obtained from BC_normal, 91,405,984 from BC_yellow, 86,447,180 from F_normal, and 79,484,452 from F_yellow. The GC contents of the sequences of the four libraries were all approximately 47%, and all Q30% scores (reads with average quality scores >30) were >90%, indicating that the accuracy and quality of the sequencing data were sufficient for further analysis. The sequenced reads were aligned to the *B. oleracea* genome reference (TO1000) (http://plants.ensembl.org/Brassica_oleracea/Info/Index) (accessed on 5 May 2017). An overview of the sequencing process is shown in Supplementary Table S3. The density distribution and boxplot of all the genes exhibited similar patterns among the four samples, indicating that no bias occurred in the construction of the cDNA libraries (Supplementary Figure S2).

**Figure 2:**
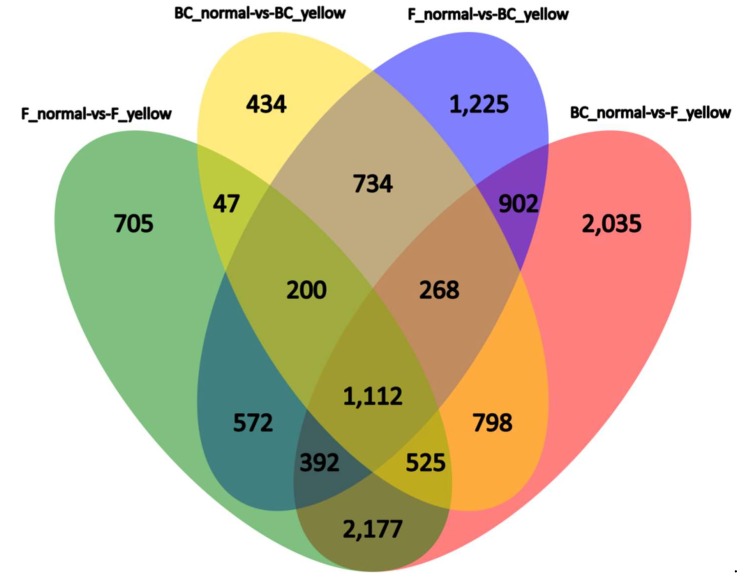
Venn diagram showing the numbers of overlapping and nonoverlapping DEGs (FDR < 0.01 and fold change > 2.0 or < −2.0) in the indicated segments from normal-leaf samples and yellow-leaf samples.

The number of DEGs identified between the yellow-green-leaf and normal-leaf samples is shown in Table 2 (Supplementary Figure S3). In the yellow-green-leaf pools, there were approximately 20% fewer down-regulated genes than up-regulated genes. In total, 5730 and 4118 (4924 on average) DEGs were detected between the yellow-green-leaf and normal-leaf pools for the F_3_ and BC_2_ populations. As shown in the Venn diagram presented in Figure 2, 1884 common DEGs were shared between the DEGs identified in BC_normal vs. BC_yellow and the DEGs identified in F_normal vs. F_yellow, representing approximately half of the total number of DEGs in either population. Cross-comparison showed that only 1112 DEGs (Supplementary Table S4) were common between yellow-leaf and normal-leaf bulks. Thus, BSR analysis using four pools greatly reduced the number of DEGs from 4924 to 1112.

**Figure 3:**
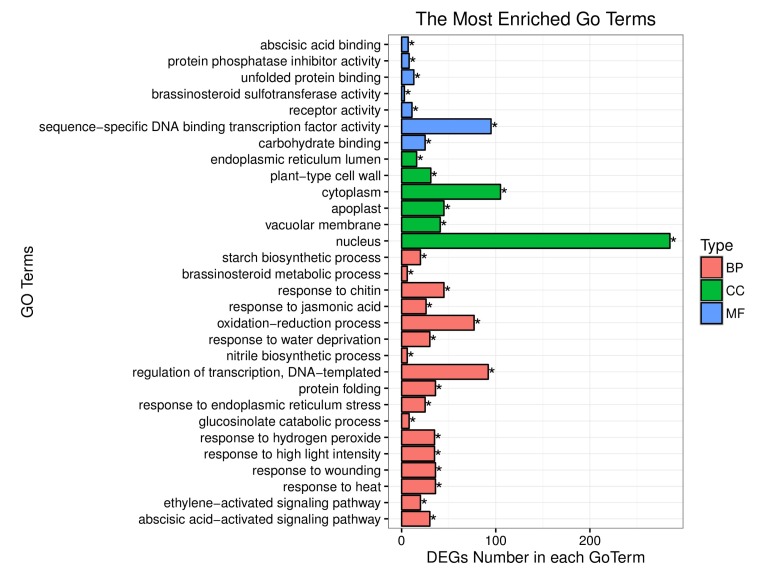
The thirty top GO assignments of 1112 DEGs. Blue: molecular function, green: cellular component, and red: biological process. The Y-axis represents the GO Term; the X-axis represents the number of DEGs for each GO Term. “*” indicates significant enrichment of the GO Term.

**Figure 4:**
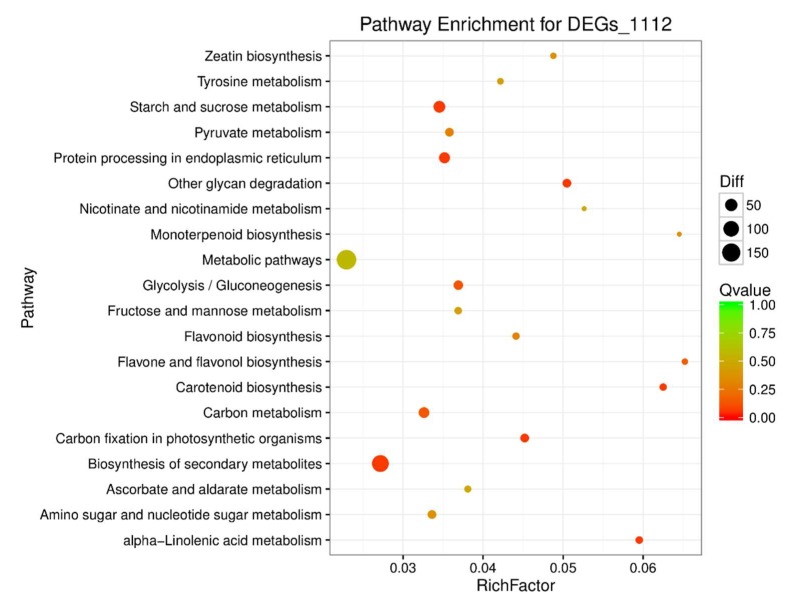
The top-20 enriched KEGG pathways of the 1112 DEGs. The Y-axis represents the pathway term; the X-axis represents the rich factor. The sizes of the points represent different DEG numbers, such that the bigger the point, the greater the DEG number. The colors represent different *Q*-values.

These 1112 DEGs were assigned into three Gene Ontology (GO) classes: biological process, cellular component, and molecular function. Thirty of the most significantly enriched of GO terms are shown in Figure 3, including “carbohydrate binding”, “sequence-specific DNA binding transcription factor activity”, “receptor activity”, “brassinosteroid sulfotransferase activity”, “unfolded protein binding” and “protein phosphatase inhibitor activity” under GO molecular functions and “endoplasmic reticulum lumen”, “plant-type cell wall”, “cytoplasm”, “vacuolar membrane”, “apoplast”, and “nucleus” under GO cellular components. Seventeen biological function or functional groups were enriched in the GO biological process category. In certain biological functions, genes play roles by interacting with each other, and KEGG pathway analysis helps provide an in-depth understanding of the biological functions of genes. A total of 1112 DEGs were annotated in the KEGG database, and 117 KEGG pathways were assigned. These 117 pathways were divided into three levels. Level one included “genetic information processing”, “metabolism”, “cellular processes”, “organismal systems”, and “environmental information processing.” The nineteen terms in level two are shown in Figure 4.

### 4.3. DEGs Involved in B. oleracea Chlorophyll Synthesis

The chlorophyll a, chlorophyll b, and total chlorophyll contents of the yellow-green-leaf mutant YL-1 were significantly lower than those of wild-type plants over the entire growth period [10]. Among the 1112 identified DEGs, 18 DEGs related to chlorophyll were clustered, which are shown in Supplementary Figure S4, including nine down- and nine up-regulated DEGs. These 18 DEGs were distributed among different chromosomes. Among the nine chromosomes, there were more DEGs on C01, C03, and C06 than on the other chromosomes (Supplementary Figure S5). In the 11.47 Mb recombination suppression region, two genes *Bo1g088040* (homologous gene *AT1G58290*, *HEMA1*) and *Bo1g098190* (homologous gene *AT1G61520*, *LCA3*) were related to chlorophyll according to the annotations, but there were not DEGs among these four pools by transcriptomics analysis and semi-quantitative PCR. Besides, no sequence variation was detected in the CDS region of these two genes of YL-1, compared with the sequences of 01-20, 11-192, and reference genome TO1000. 

**Figure 5:**
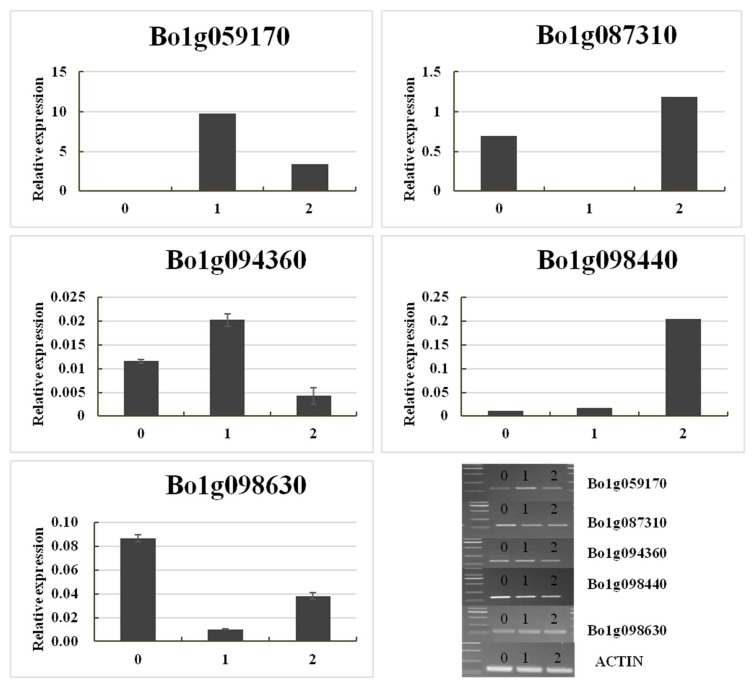
qRT-PCR and RT-PCR validation of transcripts of five DEGs associated with the yellow-green-leaf mutant. 0: the parent 01-20, 1: Mutant YL-1, 2: the parent 11-192.

DEGs located in the *ygl-1* mapping interval with recombination suppression were selected for further analysis. In the BC_normal vs. BC_yellow comparison, 82 DEGs were found in the 11.47 Mb genomic region, with 45 DEGs being down-regulated and 37 being up-regulated. In the F_normal vs. F_yellow comparison, 105 DEGs were found in the 11.47 Mb genomic region, with 47 DEGs being down-regulated and 58 being up-regulated. Among these four pools, 43 common DEGs were present, with 20 DEGs being down-regulated and 23 being up-regulated (Supplementary Table S5). According to the annotations, five of these genes were related to chloroplasts (Table 3), including the down-regulated genes *Bo1g087310*, *Bo1g094360,* and *Bo1g098630* and the up-regulated genes *Bo1g059170* and *Bo1g098440*. These five genes were applied in qRT-PCR and RT-PCR analyses of the three parents (01-20, YL-1, 11-192). The relative normalized expression of these five genes is shown in Figure 5. The primers of qRT-PCR were supplied on Supplementary Table S6. Based on the relative normalized expression, it can be observed that the expression of *Bo1g059170*, *Bo1g087310*, and *Bo1g094360* genes was consistent with the results of BSR, whereas the relative expression of the *Bo1g098440* and *Bo1g098630* genes differed from the results of BSR. We inferred that these two genes’ transcription levels were irrelevant to the yellow-green-leaf trait. In the other three genes that related to chloroplasts, *Bo1g087310* (homologous gene *AT1G56340*, Calreticulins-1) plays important roles in calciumion binding, plant growth, and plant height [33]. *Bo1g059170* (homologous gene *AT3G51420*) is involved in strictosidine synthase activity and plant defense [34], and *Bo1g094360* (homologous gene *AT3G08840*) functions in d-alanine-d-alanine ligase activity (Table 3) [35]. Hence, we inferred that these three candidate genes (*Bo1g094360*, *Bo1g087310*, and *Bo1g059170*) may be responsible for the development of the yellow-green-leaf mutant phenotype.

## 5. Discussion

### 5.1. Efficiency of BSR in DEG Detection

BSA (an efficient method for rapidly identifying markers linked to mutant phenotypes) combined with RNA-seq has been performed to map important agronomic traits at the transcription level in some species, such as catfish [23], onion [36] maize [37], Chinese cabbage [38], Chinese wheat cultivar [39], polyploid wheat [40], etc. Using BSR, Kim et al. [35] identified the candidate gene, AcPMS1, which is involved in DNA mismatch repair, for the fertility restoration of cytoplasmic male sterility in onions. Ramirez-Gonzalez et al. [24] mapped *Yr15* to a 0.77-cM interval in hexaploid wheat using a segregated F_2_ population through BSR. In the present study, RNA-seq analysis of four bulks detected only 1112 common DEGs between the four pools (4924 on average), which can reduce the number of genes related to the phenotype. Therefore, BSR was further demonstrated to be an efficient method for analyzing the genes associated with the yellow-green-leaf mutant phenotype.

### 5.2. DEGs Analysis Associated with the Yellow-Green-Leaf in a Recombination-Suppressed Region via RNA-Seq

In recent years, the fine mapping of important agronomic traits in *Brassica* has developed rapidly [41,42,43]. Some yellow leaf color genes have been mapped in *Brassica* crops. A mutation responsible for chlorophyll deficiency in *Brassica juncea* was mapped between amplified fragment length polymorphism (AFLP) markers EA4TG4 and EA7MC1, with genetic distances of 33.6 and 21.5 cM, respectively [44]. In *B. napus*, Wang et al. [45] mapped the *CDE1* locus to a 0.9 cM interval of chromosome C08, and Zhu et al. [3] mapped a chlorophyll-deficient mutant between the markers BnY5 and CB10534, which are closely linked to the chlorophyll deficiency gene *BnaC.YGL*, with genetic distances of 3.0 and 3.2 cM on C06, respectively. Gene mapping for the above leaf color mutant was based on normal recombination in the segregated population. Recombination suppression was reported in many species, such as tomato [46], barley [47], petunia [48], *Populus* [49], hexaploid wheat [50], and buffelgrass [51]. In this study, we identified a large recombination suppression region spanning ~11 Mb on C01. However, recombination rate of *Brassica oleracea* C01 in previous studies seemed to be normal. The genetic map was constructed based on *Brassica oleracea* re-sequencing data; the C01 linkage groups spanned 97.59 cM, with an average distance of 1.15 cM between neighboring loci; and no recombination suppression was found [52]. Lv et al. (2016) [53] constructed a high-density genetic map while describing a comprehensive QTL analysis of key agronomic traits of cabbage. On C01, twelve markers existed between the markers Indel481 (17,365,179 bp) and Indel14 (28,513,070 bp), which showed recombination was observed to be normal at the 17.3–28.5 Mb. In the present study, recombination suppression was observed at C01: 18,069,792–29,537,314 bp in the mapping of *ygl-1* gene using the population constructed from YL-1 and 01-20. Moreover, a recombination-suppressed region was identified in the same area while mapping *ygl-1* using another population constructed from YL-1 and 11-192. These two populations have one same parent YL-1. Therefore, we speculated that the suppression of recombination may be due to the YL-1 mutant.

In the recombination-suppressed region, it is difficult to identify candidate genes using fine mapping. Some research has revealed genes related to the phenotype by RNA-seq, such as *Fhb1* in wheat [54] and *BPH15* in rice [55]. In the *ygl-1* gene-mapping interval, a total of 10478 SNPs and Indels, with 455 genes, were identified in the recombination-suppressed region, including 78 genes related to chloroplasts. Comparison of the two bulk RNA-seq groups showed that only 43 genes were common DEGs, only five of which were related to chloroplasts. Furthermore, three of these five genes’ expression by qRT-PCR were consistent with the results of BSR. Therefore, BSA combined with RNA-seq was able to greatly reduce the number of DEGs, demonstrating that this method is an effective alternative for identifying candidate genes in a recombination-suppressed region. 

### 5.3. Assembly Error in the Reference Genome

*Brassica oleracea* reference genome sequencing was completed in 2014 [25,56]. However, the 02-12 reference genome assemblies have been woefully incomplete, and some assembly errors have been identified in recent studies. For example, Lee et al. [47] revised 27 v-blocks, 10 s-blocks, and several other blocks in the 02-12 reference genome assembly during the mapping of clubroot resistance QTLs through genotyping-by-sequencing. The purple leaf gene (*BoPr*) in the ornamental kale was mapped on an unanchored scaffold by Liu et al. (2017) [57]. In a previous study [7], we identified possible assembly errors in the 02-12 reference genome. According to the comparison of marker positions with the TO1000 reference, the physical locations of Part II and Part III in the 02-12 reference genome likely represent assembly errors (Figure 1). The makers’ order of linkage map was consistent with the physical map order of TO1000 reference genome. All the results showed that the TO1000 reference genome is reliable. These results will contribute to the improvement of the cabbage genome.

## 6. Conclusions

In conclusion, we mapped the yellow-green-leaf gene *ygl-1* on a recombination-suppressed genomic region by two populations. Bulk segregant RNA-seq (BSR) was applied to identify differentially expressed genes using two segregate populations. BSR analysis with four pools greatly reduced the number of common DEGs from 4924 to 1112. Eighteen DEGs related to chlorophyll were clustered. In the *ygl-1* gene mapping region with suppressed recombination, 43 common DEGs were identified. Five of the genes were related to chloroplasts; the *Bo1g098440* and *Bo1g098630* genes were excluded by qRT-PCR. Hence, *Bo1g059170*, *Bo1g087310*, and *Bo1g094360* in the mapping interval may be tightly associated with the development of the yellow-green-leaf mutant phenotype. Further studies on these genes may reveal the molecular mechanism of yellow-green-leaf formation in *B. oleracea.*

## Figures and Tables

**Table 1:** Genetic distances of the InDel primers to the *ygl-1* in the two mapping populations.

YL-1 × 01-20		YL-1 × 11-192	
Primers	Genetic Distance (cM)	Primers	Genetic Distance (cM)
T2-3	9.21	T2-3	13.3
T2-5	6.90	-	-
T1-1	6.28	-	-
T1-14	4.39	T1-14	6.5
T1-18	3.97	T1-18	4.4
T1-26	2.51	T1-26	2.3
T1-28	1.46	T1-28	1.5
T1-30	1.05	T1-30	1.3
T1-34	0.63	T1-34	0.3
T1-36	0.42	T1-36	0.00
T1-58	0.42	T1-58	0.7
T2-6	0.42	T2-6	1.04
T2-10	0.63	T2-10	1.04
T2-14	0.63	T2-14	1.04
T2-16	3.14	T2-16	2.61
T2-18	5.02	T2-18	6.02

**Table 2:** Numbers of DEGs between the yellow-leaf and normal-leaf samples.

	No. of DEGs	No. of Up-Regulated DEGs	Percentage (%)	No. of Down-Regulated DEGs	Percentage (%)
BC_normal vs. BC_yellow	4118	2384	58	1734	42
BC_normal vs. F_yellow	8009	4894	60	3315	40
F_normal vs. F_yellow	5730	3226	56	2504	44
F_normal vs. BC_yellow	5405	2844	53	2561	47

**Table 3:** DEGs related to chloroplasts in the recombination-suppressed region.

Gene ID ^a^	Physical Distance (TO1000)	F Normal ^b^	F_Yellow ^b^	BC_Normal ^b^	BC_Yellow ^b^	Diff ^c^	A.T. Annotation ^d^
Bo1g087310	C1:25381300–25383803	1837.98	156.85	1920.64	287.42	Down	Calreticulins-1, response to oxidative stress, response to cadmiumion, response to salt stress, calciumion homeostasis;
Bo1g094360	C1:27829353-27834745	48.65	10.53	29.70	2.04	Down	d-alanine-d-alanine ligase activity
Bo1g098630	C1:29261755-29263303	4002.89	475.81	1119.81	125.36	Down	GPT2: glucose-6-phosphate/phosphate translocator 2
Bo1g059170	C1:18110687-18112080	167.35	828.45	277.80	858.19	Up	SSL4: strictosidine synthase-like 4
Bo1g098440	C1:29037892-29038492	129.41	285.27	120.70	427.05	Up	Protein of unknown function, DUF538

^a^ Five *B. oleracea* DEGs related to chloroplasts (reference genome TO1000). ^b^ Expression levels in the four samples. ^c^ Differential regulation: up-regulation and down-regulation. ^d^ GO annotations for seven Bo to AT best-hit genes obtained from The Arabidopsis Information Resource (TAIR).

## Supplementary Materials

Supplementary materials can be found at http://www.mdpi.com/1422-0067/19/10/2936/s1.
