# Supplementary material for: Differentially Expressed Genes Associated with the Cabbage Yellow-Green-Leaf Mutant in the ygl-1 Mapping Interval with Recombination Suppression
Source: Int J Mol Sci. 2018 Sep 27;19(10):2936. doi: 10.3390/ijms19102936 (PMC6212964; doi:10.3390/ijms19102936)
Supplement: Supplementary file 1 [file ijms-19-02936-s001.zip › Supplementary material/Supplementary Table 1.docx]

| Primer name | Physical distance (bp)  (02-12) | Physical distance (bp) ( TO1000) | Forward primer sequence  (5′-3′) | Reverse primer sequence  (5′-3′) |
| --- | --- | --- | --- | --- |
| BCYM475 | C1:11563641 | C1:12290344 | GCAGAACATTCTCATTCAAG | AGATGCTCCTATGTTGCATT |
| BCYM516 | C1:12317371 | C1:12979415 | ACTGTGGCTAATATTGTGGG | TACCACACTAAGACAACGCA |
| BCYM543 | C1:13287495 | C1:13645294 | CCTTGATCCTCTTGTTGTTC | ACCATTTGAGTTTGTACCTG |
| BCYM577 | C1:14228547 | C1:14521615 | TGTCAGTCCTTCCAGGTAGA | GGCCATCAACTTCACTAAAC |
| BCYM585 | C1:14547932 | Scaffold00751:49675 | TAGACCAGCTTGAGTAAGCC | GAATGCAGGTATCGTCTGTT |
| BCYM593 | 15700975 | [C1:21177688](http://plants.ensembl.org/Brassica_oleracea/Location/View?r=C1:21177687-21177707;tl=MdmtfLDPDAV3sS0R-15722343-231526310) | TTGACTACAATGCTGTCTGC | CTAAATTGCTCCATTCCTTG |
| BCYM611 | 16404614 | C1:21765777 | TGTTTCCTCGCTAATTCCTA | CAGGGTTTAGTCTGTTTTGG |
| BCYM622 | 16710365 | C1:22035203 | GGCAAAGGATAAATAGGGAT | GTTCTGCTTTGATTCCTACG |
| BCYM643 | 17339297 | C1:23143013 | TTTAACCGGATTAGACCAGA | ATACCTTCCGATGTTCCATA |
| BCYM662 | 17827247 | C1:23608274 | ATGACACACTTGCTTGATGA | ACCATTTCTAGACACGGAAA |
| BCYM706 | 20128235 | C1:25712629 | GTGTCCGTTAATGGAGTTGT | GGATGATGGGGGATAAAAT |
| BCYM726 | 20640696 | C1:26201935 | TCAGTCTTGATTGTTGTTGC | CCTGATCCTTTGCAATCTAA |
| BCYM804 | 23353865 | C1:29307981 | AGTACGGGAACGACAAGTTA | CTTCAAAATCACCAACAAGG |
| BCYM825 | C1:24060605 | C1:19230187 | CAACAAGGTAAGAGTCTGGG | GTCACACACAGAAACAACTAGG |
| YL135 | C1:24372012 | C1:17191806 | TTCTTCCCACAAAACCTTC | TATGCCCCAACATTAAAGC |
| BCYM841 | C1:24476234 | C1:17298977 | AGCTAAAATCATGGAGAGTG | TTGATAACCTTTGTGGAACC |
| BCYM851 | C1:24898206 | C1:17461534 | CTTTCACAGATTTGATGTGG | AAAAACCTGACCCGAATC |
| ID2 | C1:25357762 | C1:18126217 | TCCGACAACGAAGATTTCA | TGCAGTTATTCGGTGGAGA |
| M8 | C1:25524704 | C1:29537261 | TCAGGCAGCAAATAGTAGAGAG | AATCAGGTCCAATGCGTC |
| BCYM873 | C1:25706570 | C1:29759609 | CTCATGATTACTTGGCAACA | TCGTCTTACGTTACGTTCCT |
| BCYM886 | C1:26599434 | C1:30564487 | GTAAACCGTAAAATCATGCC | CGGTTATGGAAGGAAAATGT |
| BCYM890 | C1:27331406 | C1:30873431 | CCGTGAACAAATAGGAAAGA | GGCAAAGGGAATGATATTAG |
| BCYM935 | C1:29306904 | C1:33197656 | TTCAAAATGGTGGGACTTAG | CTCATATTTCTTTTCGCCTG |
| BCYM941 | C1:29620770 | C1:33464302 | GTTGCGGAGAAGGAGAAA | GAAAACAGCTTATTCATGCC |
