# Supplementary material for: Differentially Expressed Genes Associated with the Cabbage Yellow-Green-Leaf Mutant in the ygl-1 Mapping Interval with Recombination Suppression
Source: Int J Mol Sci. 2018 Sep 27;19(10):2936. doi: 10.3390/ijms19102936 (PMC6212964; doi:10.3390/ijms19102936)
Supplement: Supplementary file 1 [file ijms-19-02936-s001.zip › Supplementary material/Supplementary Table 2.docx]

| **Primer name** | **Forward (5’-3’)** | **Reverse (5'-3')** |
| --- | --- | --- |
| T2-3 | aaacagcctgcagatacactatga | ttcctctgttttatcattgcttca |
| T2-5 | gaagtctaggtactgtgttagttgcg | caaggactgtaaaatgctgctaga |
| T1-1 | aggtttctctcctcttgtgattgt | aaagccatctagagataaaagcca |
| T1-14 | gcatgatcagcgtcttgtacttat | cgctgacacaaaaatatctaaacaa |
| T1-18 | gttggttaacattcacacgacaat | catttggttagacgaaaatgtgac |
| T1-26 | gtctcttgaaatgtgtgttgctct | agcaaaacttgaaacgcttcttat |
| T1-28 | ttttggaagtatctgaccaaatca | agatgagagagaggaggatgagc |
| T1-30 | tgaaaattttgttgttcaagagga | cttgccactcaatatccagactta |
| T1-34 | gaatctctcgtgttcttcaaggtt | ttgaaatttctcagactcaaaacg |
| T1-36 | ttgaagcgtctagtgtttgaaaat | tttttccacttttcttttaatccg |
| T1-58 | tcaggcagcaaatactagagagtg | gactcatgaaccacagactctgac |
| T2-6 | agatattgagttcgggtctctttg | agatcagcattgaactttgcatta |
| T2-10 | tgaaagacgattgcttctttacac | tttttaaactgaaccattctgcaa |
| T2-14 | aagcagactcagtagtcagcagaa | catggacttctacaatcagaaacg |
| T2-16 | ttttgaacaattgaaaagggaaat | ggtggagtttgaaaccagatacat |
| T2-18 | gcataaccataaagcctaagcaat | ttttagggtttgagcaaagttagg |
