# Supplementary material for: Differentially Expressed Genes Associated with the Cabbage Yellow-Green-Leaf Mutant in the ygl-1 Mapping Interval with Recombination Suppression
Source: Int J Mol Sci. 2018 Sep 27;19(10):2936. doi: 10.3390/ijms19102936 (PMC6212964; doi:10.3390/ijms19102936)
Supplement: Supplementary file 1 [file ijms-19-02936-s001.zip › Supplementary material/Supplementary Table 3.docx]

| Type | BC_normal | BC_yellow | F_normal | F_yellow |
| --- | --- | --- | --- | --- |
| Read Length | 150 | 150 | 150 | 150 |
| Total Raw Reads | 82143852 | 91405984 | 86447180 | 79484452 |
| Total Raw Bases | 12321577800 | 13710897600 | 12967077000 | 11922667800 |
| Total Clean Reads | 81742194 | 91023634 | 86085980 | 79053354 |
| Total Clean Reads Ratio(%) | 99.51 | 99.58 | 99.58 | 99.46 |
| Total Clean Bases | 12261329100 | 13653545100 | 12912897000 | 11858003100 |
| Total Clean Bases Ratio(%) | 99.51 | 99.58 | 99.58 | 99.46 |
| Total Adatper Reads | 399972 | 380588 | 359486 | 429690 |
| Total Adatper Reads Ratio(%) | 0.49 | 0.42 | 0.42 | 0.54 |
| Total Low Quality Reads | 1686 | 1762 | 1714 | 1408 |
| Total Low Quality Reads Ratio(%) | 0 | 0 | 0 | 0 |
| Clean Reads GC(%) | 47.36 | 47.09 | 47.31 | 46.76 |
| Clean Reads Q20(%) | 97.22 | 97.28 | 97.15 | 97.32 |
| Clean Reads Q30(%) | 93.11 | 93.25 | 92.97 | 93.34 |
| Total_mapped_reads | 78.41% | 77.89% | 78.54% | 77.40% |
