# Supplementary material for: Differentially Expressed Genes Associated with the Cabbage Yellow-Green-Leaf Mutant in the ygl-1 Mapping Interval with Recombination Suppression
Source: Int J Mol Sci. 2018 Sep 27;19(10):2936. doi: 10.3390/ijms19102936 (PMC6212964; doi:10.3390/ijms19102936)
Supplement: Supplementary file 1 [file ijms-19-02936-s001.zip › Supplementary material/Supplementary Table 6.docx]

| Primer name | Forward primer sequence  (5′-3′) | Reverse primer sequence  (5′-3′) |
| --- | --- | --- |
| Bo1g059170 | TGTCGTAGAGAACTGGGTCA | CCGTCAACAACTCCGTCTT |
| Bo1g087310 | ATCAACAAGGAGGTCCCA | TCCCAGTCAGAGTAGAGGCT |
| Bo1g094360 | ATGGATGGTATTTGACTCCC | CCTTAGAAGCCTGCTGGAA |
| Bo1g098440 | GGTTACCAGTCAAAGGCATT | TGGATAGCAAACAGGAGGA |
| Bo1g098630 | CTTCCCGACGAACAATCA | CACAAGCGAGAGACAAGGT |
| ACTIN | TCCAATCTACGAGGGTTT | AGTCTCCATCTCCTGCTC |
