# Supplementary figures and images for: Differentially Expressed Genes Associated with the Cabbage Yellow-Green-Leaf Mutant in the ygl-1 Mapping Interval with Recombination Suppression
Source: Int J Mol Sci. 2018 Sep 27;19(10):2936. doi: 10.3390/ijms19102936 (PMC6212964; doi:10.3390/ijms19102936)

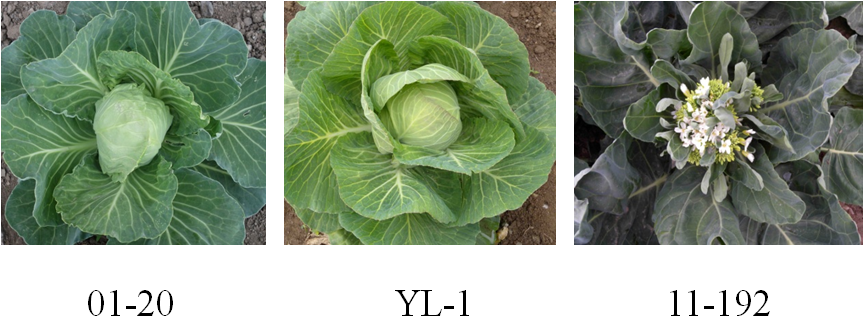

Supplement: Supplementary file 1 [file ijms-19-02936-s001.zip › Supplementary material/Supplementary Figure 1.png]

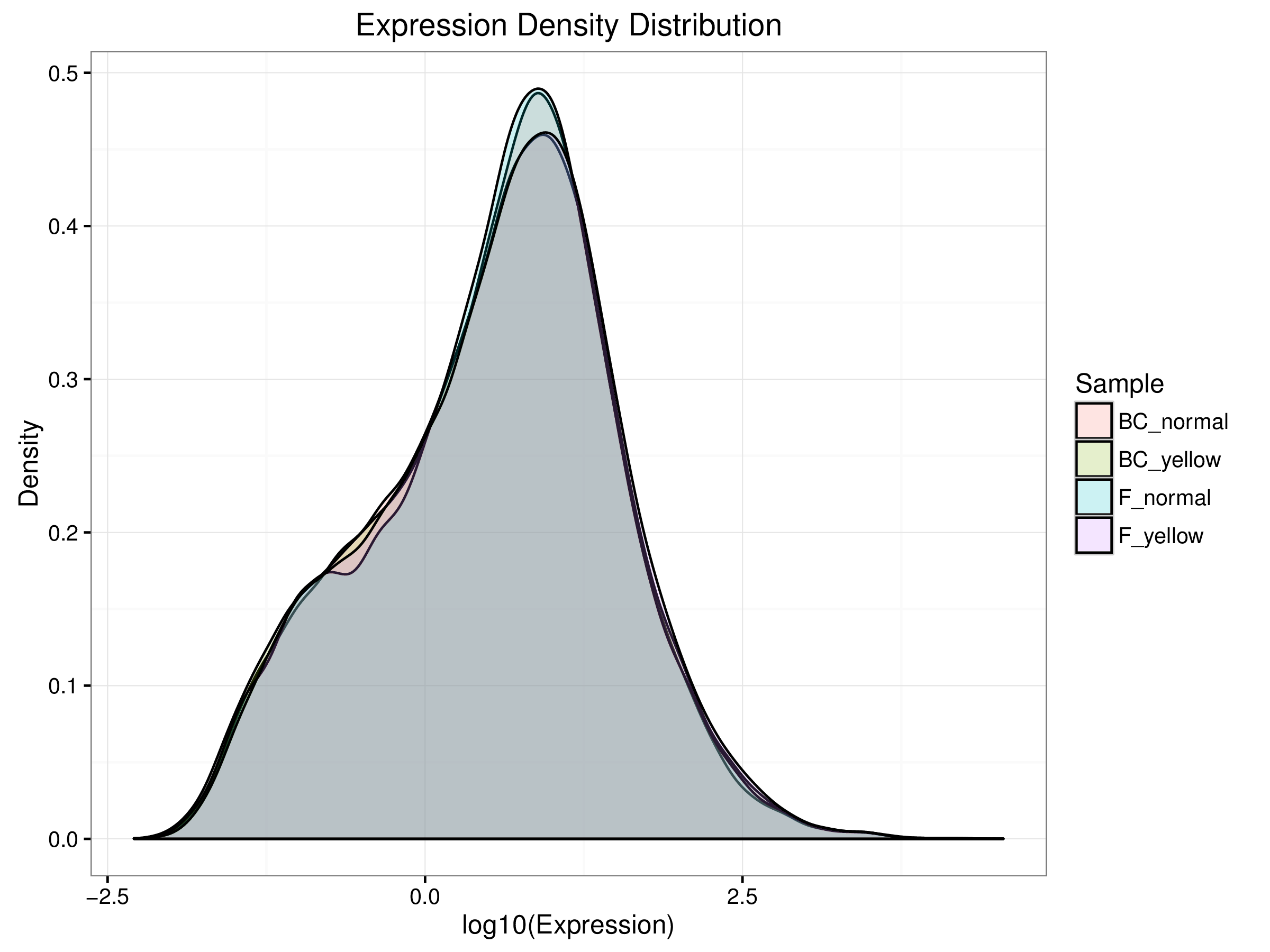

Supplement: Supplementary file 1 [file ijms-19-02936-s001.zip › Supplementary material/Supplementary Figure 2.png]

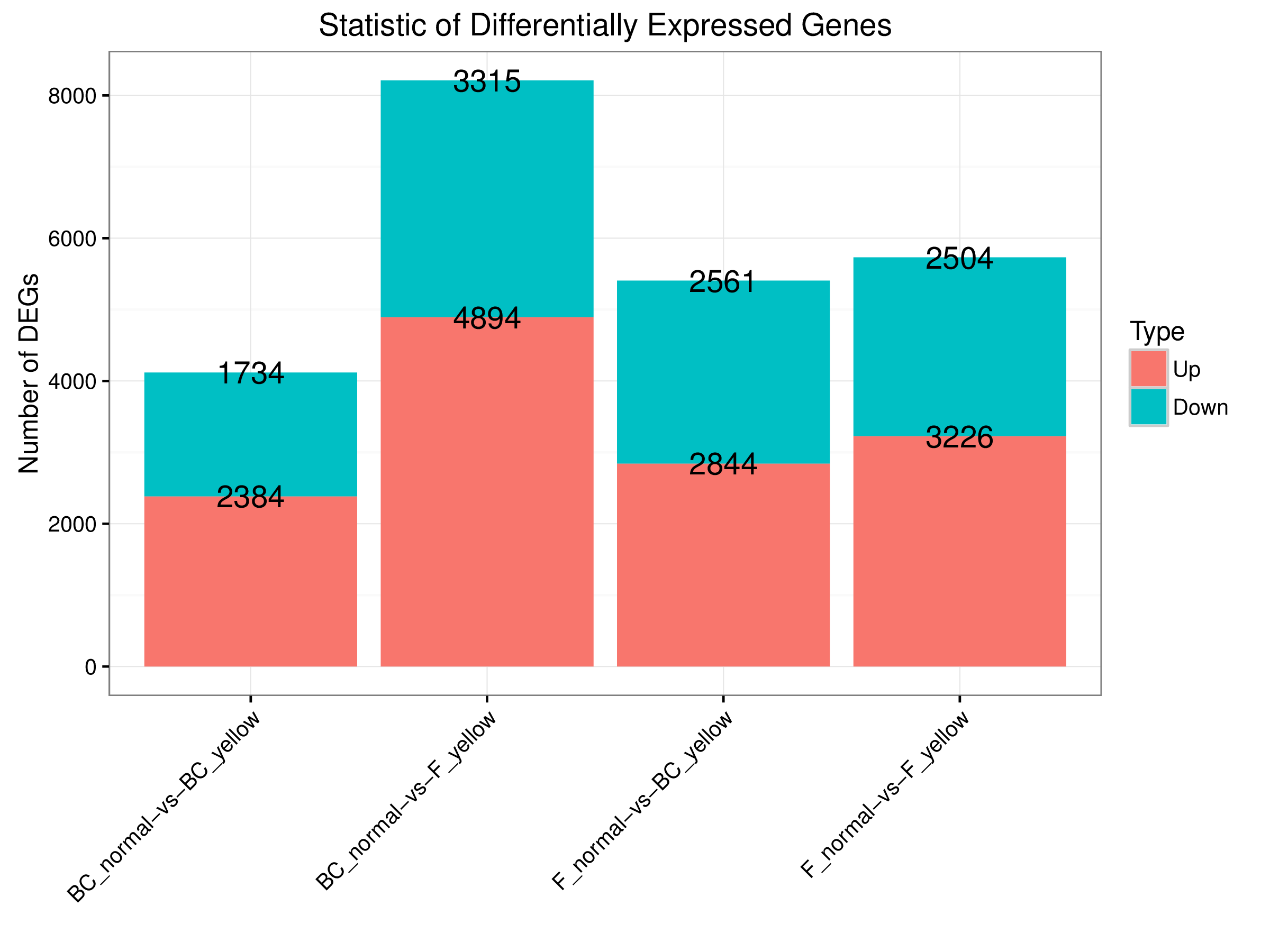

Supplement: Supplementary file 1 [file ijms-19-02936-s001.zip › Supplementary material/Supplementary Figure 3.png]

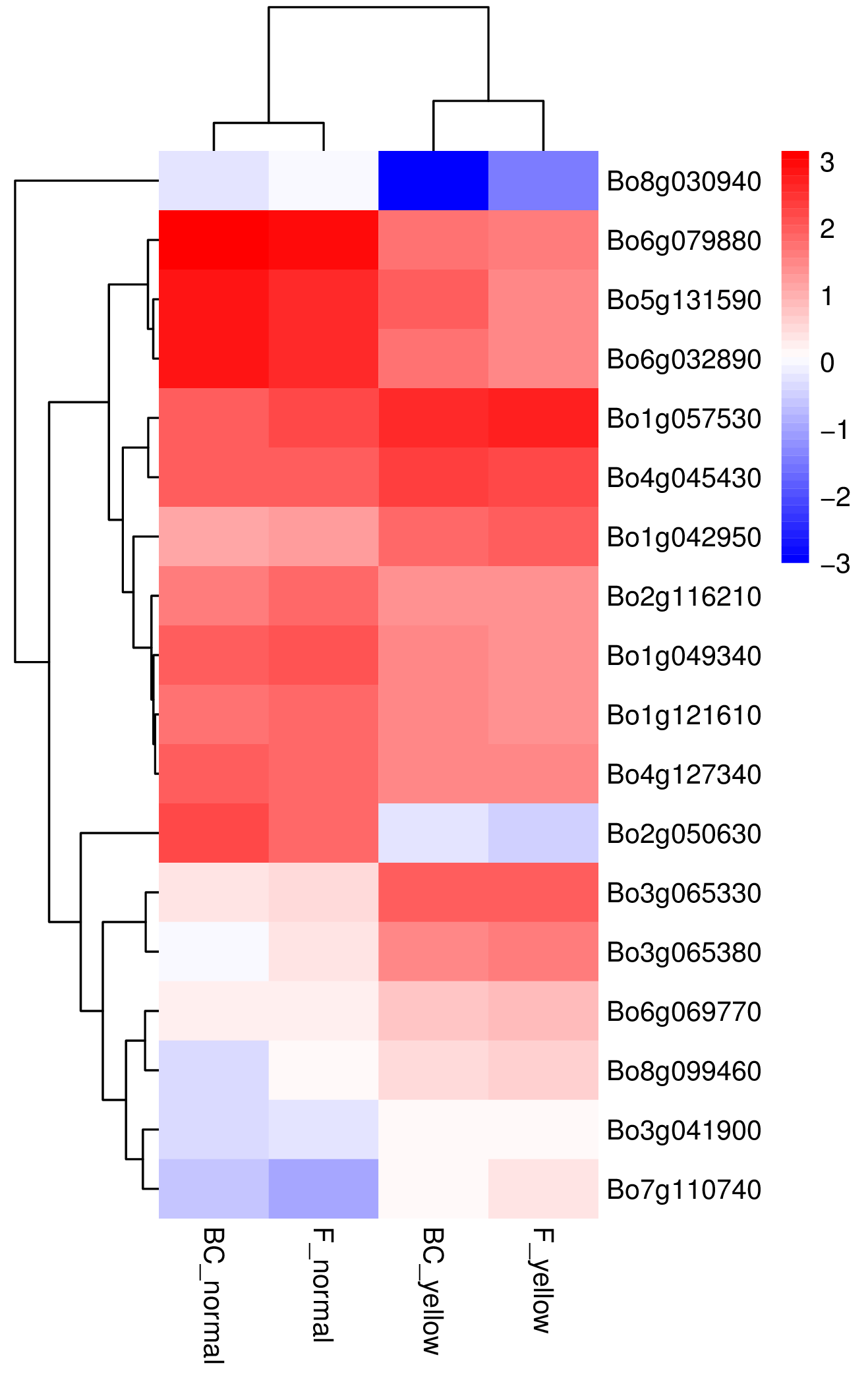

Supplement: Supplementary file 1 [file ijms-19-02936-s001.zip › Supplementary material/Supplementary Figure 4.png]

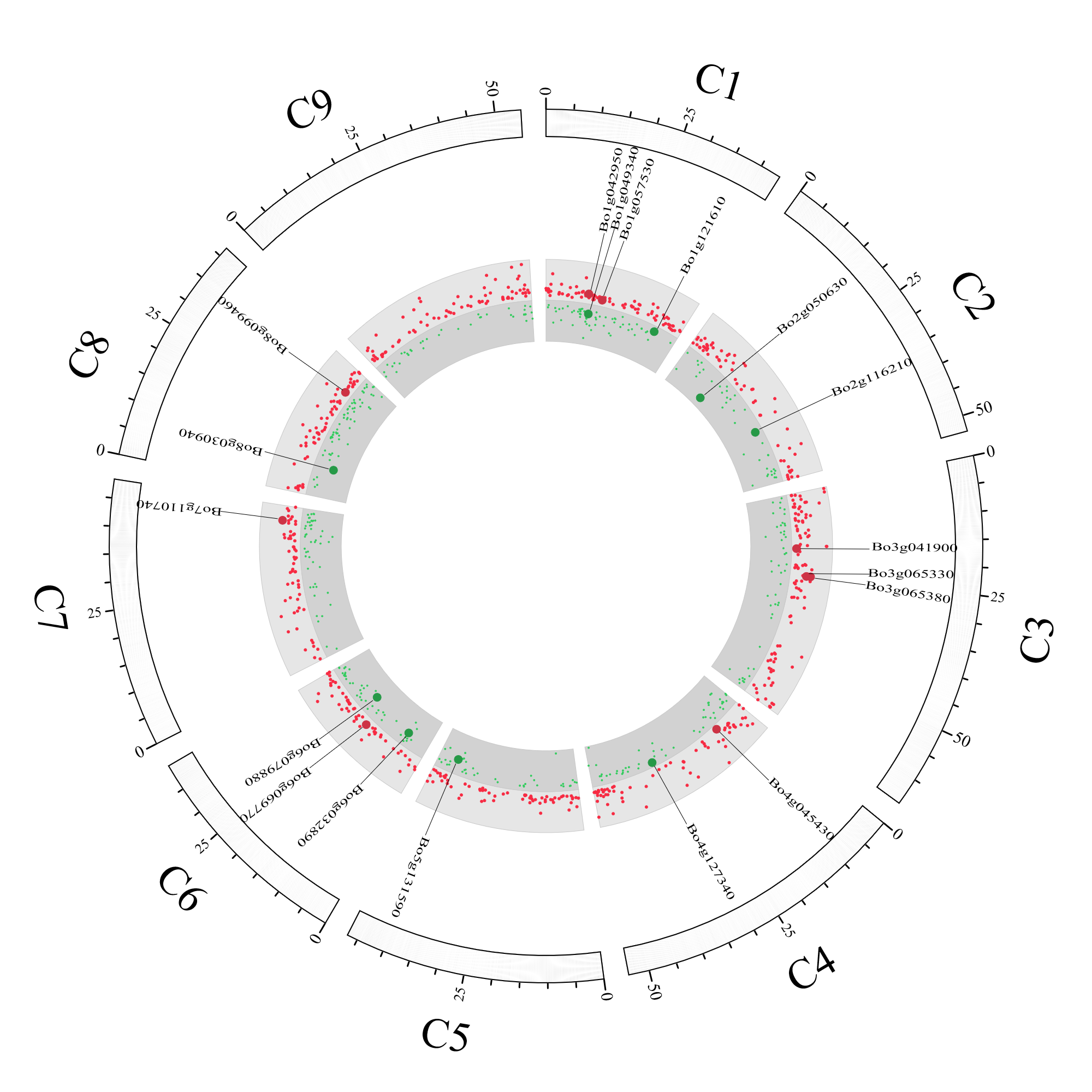

Supplement: Supplementary file 1 [file ijms-19-02936-s001.zip › Supplementary material/Supplementary Figure 5.png]
